# Health care workers’ knowledge and perceptions on WHO hand hygiene guidelines, and the perceived barriers to compliance with hand hygiene in Cyprus

**DOI:** 10.1186/s12912-024-02181-8

**Published:** 2024-09-11

**Authors:** Despo Constantinou, Ioannis Leontiou, Meropi Mpouzika, Koralia Michail, Nikos Middletton, Anastasios Merkouris

**Affiliations:** 1 Department of Nursing, Cyprus University of Technology Limassol Cyprus, Nicosia, Cyprus; 2 A&E Nicosia General Hospital, Nicosia, Cyprus

**Keywords:** Hand hygiene, Five (5) moments, Perception or attitude, Knowledge or understanding, Health Care professionals or health worker, Hospital or acute setting, Barriers, WHO guidelines

## Abstract

**Background:**

Hand hygiene (HH) is recognized as an important measure to avoid the transmission of harmful germs, and assists significantly in preventing healthcare-associated infections. HH compliance among health care workers (HCWs) is a result of their knowledge and perceptions.

**Aim:**

To investigate the knowledge and perceptions of WHO hand hygiene guidelines among HCWs, and the perceived barriers to compliance with hand hygiene in a major public hospital in Cyprus.

**Methods:**

A descriptive correlational study was conducted in September of 2019. The target population was all of the HCWs in Nicosia General Hospital (*N* = 1,386). The final sample consisted of 820 participants (119 physicians, 613 nurses, 27 physiotherapists, 59 ward assistants, 2 unidentified). This study used the HH knowledge and perception questionnaire that was developed by the WHO.

**Results:**

The results revealed that the average percentage score for knowledge among our sample was 61%, and statistically significant differences were observed among HCWs with regard to certain questions. It was found that HCWs, in most of their responses, presented high percentages of correct answers regarding their perceptions on hand hygiene guidelines but several perceived barriers to compliance on HH guidelines were identified as well.

**Conclusions:**

Knowledge and perceptions of HH guidelines among HCWs were moderate and good respectively. In addition, several perceived barriers to compliance on HH recommendations were identified. HH education is recognized as an important tool for removing these barriers but the recommended HH strategy should be multi-modal and consider local resources, administrative support and barriers to compliance with HH.

## Background

Healthcare-associated infections (HCAI) are a serious global public health issue [1], as they prolong the duration of hospitalization, reduce quality of life, increase antimicrobial resistance (AMR) [2], and increase morbidity and mortality [3]. It is estimated that more than 1.4 million people are suffering from HCAI worldwide and according to the WHO, 5–15% of patients admitted to a hospital unit develop at least one HCAI [4, 5].

Infection prevention and control (IPC) is a core component of safety programs for patients all around the world [6]. A report from the Organization for Economic Co-operation and Development (OECD) estimated that promoting simple IPC measures such as HH could reduce the AMR health burden by approximately 40% [7]. Increased HH performance in health care can lower HCAIs by up to 50% [8]. Implementation of HH among HCWs is recognized as a cost-effective measure to reduce HCAIs, although compliance remains on average 40–50% worldwide [9–11]. Furthermore, it is accepted that prevention of HCAIs can be fully achieved by continuous compliance with HH by HCWs [12].

The World Health Organization (WHO) guidelines published in 2009 recommend a multimodal strategy that should include routine evaluation of HH compliance as a key means of improving and maintaining compliance rates among HCWs. Also, WHO guidelines include an assessment tool of HCWs’ knowledge and perception of HH [5].

Many studies investigating factors contributing to HH compliance indicate that non-compliance is of great concern in hospital settings. HCW’s lack of knowledge regarding both HH guidelines and basic infection control measures were identified by these studies as the main issues [13–16].

The importance of our study is that it focuses on an issue that is a major public health problem worldwide, and which had not been investigated in the hospitals of Cyprus. Since there was no research-based evidence in Cyprus on the knowledge and perceptions of HCWs concerning HH guidelines, we initiated our research in order to identify and implement all necessary interventions to correct any problems that we identified. It is worth noted that the results of this study can be used as a guide for all stakeholders with the ultimate goal of improving quality of care, reducing healthcare costs, and ensuring patient health and safety.

## Methods

### Aim

The aim of this study was to investigate both the knowledge and the perceptions of HCWs of WHO HH guidelines, and the perceived barriers to compliance with HH in a major public hospital in Cyprus.

### Study design

The study used a descriptive correlational design and was conducted in September 2019 at the Nicosia General Hospital, which is the largest tertiary hospital in Cyprus. The study addressed physicians, nurses, physiotherapists and ward assistants working in all clinical departments/ wards where 24 h care was provided. The target population was all HCWs (*N* = 1,386).

### Instruments

The WHO tools *“WHO Perception Questionnaire for Health Care Workers”* and *“Hand Hygiene Knowledge Questionnaire for Health Care Workers”* (WHO, 2009) were obtained upon request through email by the WHO. The distributed combined questionnaire consisted of four domains: socio-demographic data (domain A), perception (domain B), HH knowledge (domain C), and barriers (domain D). Domains A, B and C consisted of 31 questions with sub questions (items). Domain D comprised 10 items. There were eleven items included in domain A, twelve items in domain B, twelve items in domain C, and ten items in domain D.

A systematic approach to the relevant literature was employed to examine perceived barriers to HH. A total of 20 selected papers revealed 16 barriers, of which the 10 most frequently observed (as agreed between three researchers) were selected. These 10 additional items comprised the last part of our questionnaire with the aim of exploring participants’ perceived barriers to compliance on HH. The items were measured on a 5- point scale from unimportant to extremely important.

Overall, responses to twelve of the items were rated on a 7-point scale (not effective - very effective, no importance to very high importance and no effort to a big effort). Three items were rated on a 4-point scale (very low to very high, and very low priority to very high priority). Nine items were multiple choice (coded as right answer = 1, wrong answer = 0). Four items were true or false, and 14 were yes or no. Two questions were used to assess self-reported HH performance of self and of others (other HCWs), and one was used to estimate the rates of development of a HCAI (0-100%). On average, the questionnaire took less than fifteen minutes to be completed.

### Translation process, cultural adaptation and pilot testing

Although the tool “*Hand Hygiene Knowledge Questionnaire for Health Care Workers”* was translated in Greek previously by Tsekoura et al. (2018) we perform our translation for both WHO tools described above. Bidirectional bilingual translation (forward translation and backward translation) was used to create the Greek version of the questionnaire by two different people (one for the forward translation and one for the backward) in order to test the face and content validity of the research instrument [17–19]. Subsequently it was given to four experts with expertise in infection control (two nurses and two physicians). Based on their suggestions, a second version of the questionnaire was developed by rephrasing certain statements without changing their basic meaning. This final questionnaire was piloted on 30 HCWs in order to further evaluate its content validity. When it was accepted that the Greek version was generally understood, and no further suggestions were made for improving the wording, the questionnaire was adopted for use in our study.

### Demographic characteristics

The demographic data collected were age, gender, occupational group, ward/department, and whether participants had previously attended a HH training program (Yes/No).

### Data collection

The questionnaires were distributed in the workplace by the researcher to each participant, with a request that they should be returned within two weeks. Along with each questionnaire, an envelope was provided in which the participants sealed their completed questionnaires, and then placed the sealed envelopes in a box in the office of the ward manager. In order to increase the response rate, a reminder was given one week after questionnaire distribution. The questionnaires were completed by the HCWs in their free time so as not to interfere with their duties. Initially the completed questionnaires were stored by the ward manager. Subsequently they were collected by the researcher and kept at her office. The electronic form of the data was saved on a computer and password-protected access was permitted exclusively for the researcher. An information sheet and the consent statement were attached to the front of the questionnaire, therefore informed consent was implied when participants completed and returned the questionnaires.

### Data analysis

Descriptive statistics were used to describe the participants’ characteristics. T-tests were performed to determine whether there were any significant differences between the mean scores of the various variables between the various groups e.g., men and women. Chi-square tests were performed to assess the association of the perceptions on the WHO HH guidelines and perceived barriers to compliance to HH between the various groups. One way ANOVA tests were used for correlation across the occupational groups.

The software used for performing data analysis was the Statistical Package for the Social Sciences (SPSS 25.0).

### Ethical considerations

Approval was obtained from the Cyprus National Bioethics Committee (ΕΕΒΚ ΕΠ 2018.01.120) and permission for the study was granted by the Ministry of Health. Also, permission to gain access to the hospital was granted by the Nicosia General Hospital administration. Permission to use the WHO questionnaires was obtained.

## Results

### Sociodemographic data

A response rate of 60% was obtained. Demographic data are shown on Table 1.

**Table 1:** Demographics of health professional knowledge of hand hygiene

	*N*	Valid Percentage (%)
**Age Group** *(Years)*		
< 30	182	24.2
31–40	311	41.4
41–50	157	20.9
51–60	92	12.3
61>	9	1.2
Missing System	69	
**Total**	**820**	**100.0**
**Gender**		
Men	259	31.6%
Women	560	68.4%
Missing System	1	
**Total**	**820**	**100.0**
**Profession**		
Physicians	119	14.5
Nurse	613	74.9
Physiotherapist	27	3.3
Ward Assistant	59	7.2
Missing System	2	
**Total**	**820**	**100.0**

Overall, about 45% of respondents had attended an educational program relevant to this study. The majority of physicians (77.3%), nurses (51.8%) and physiotherapists (85.2%) had not attended any infection control and prevention programs that included HH in the past 3 years. The percentage of ward assistants who had not attended any such program was much lower (30.5%). These results may suggest that hospital policy had placed more emphasis on the training of ward assistants who had no previous formal training on infection control and hand hygiene. Additionally, 90.5% of the participants were routinely using an alcohol-based handrub for HH although no statistically significant difference was observed between the use of alcohol-based handrub and (a) those who received formal training in HH in the last three years (b) men and women, (c) age.

### Levels of knowledge

**Fig. 1:**
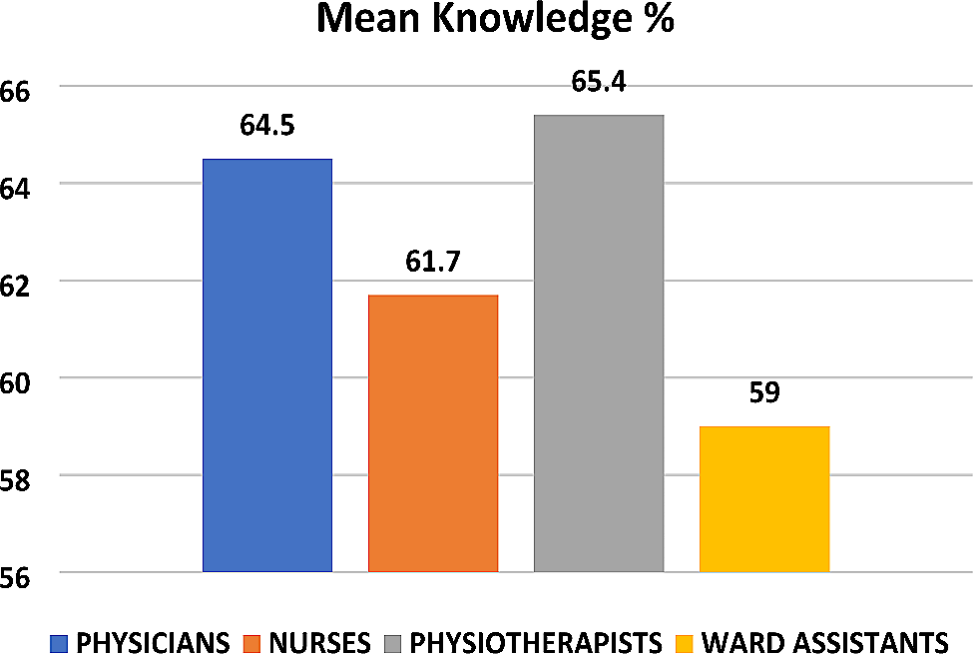
The mean percentage for knowledge scores

Mean perceived hand hygiene knowledge was calculated based on the percentage of correct responses both by occupational category and across the entire sample. The results revealed that the overall average percentage score for knowledge was 61%. Also, the results revealed that in several questions, the percentage of the sample that gave an incorrect answer was higher than the percentage of the sample that answered correctly. Furthermore, the results showed statistically significant differences in certain questions concerning knowledge among HCWs (Table 2). The highest percentage of correct answers was returned by physiotherapists (65.5%), followed by physicians (64.5%) (Fig. 1).

Mean perceived hand hygiene knowledge was calculated based on the percentage of correct responses both by occupational category and also across the entire sample (Table 2). The lower percentages of correct answers were found in the following questions:

i) *What is the most frequent source of germs responsible for HCAI?* The ward assistants presented higher rates of correct answers but this may be due to the fact that this group size was small.
ii) *HH immediately after a risk of body fluid exposure prevents transmission of germs to the patient?* The higher percentage of correct answers was found among nurses (11.5%).
iii) *Which of the following statements on alcohol-based handrub and handwashing with soap and water are true?*
  - *Hand rubbing causes skin dryness more than handwashing.*
  - *Hand rubbing is more effective against germs than handwashing.*
  - *Handwashing and hand rubbing are recommended to be performed in sequence.*
iv) *What is the minimal time needed for alcohol-based handrub to kill most germs on your hands?*

The highest percentage of correct answers across all occupational groups was found among physicians, nurses and physiotherapists on all items of the question: *Which of the following should be avoided as it is associated with increased likelihood of colonisation of hands with harmful germs?* The lowest percentage of correct answers to this question was found among ward assistants (Table 2). A possible explanation for these results is the fact that physicians, nurses and physiotherapists have better knowledge on infection control which they have gained through their academic studies.

The age group 41–50 years presented higher percentages of correct answers in all items of the questions concerning perceived knowledge, ranging from 65.4 to 83.4%, compared to other age groups. Furthermore, the HCWs who attended an infection control and prevention program that included HH in the past 3 years presented a higher percentage of correct answers than those who did not, revealing statistically significant differences.

**Table 2:** Levels of knowledge among health-care workers

Questions on Knowledge (Correct Answers)	TOTAL *N* (%)	*P*	Physician *N* (%)	Nurses *N* (%)	Physiotherapists *N* (%)	Ward Assistants *N* (%)
**Which of the following is the main route of cross-transmission of potentially harmful germs between patients in a health-care facility?** (Health-care workers’ hands when not clean)	518 (63.6)	0.552	65 (55.1)	357 (58.3)	14 (53.8)	29 (49.2)
What is the most frequent source of germs responsible for health care-associated infections? (Germs already present on or within the patient)	195 (23.9)	0.000	21 (17.8)	138 (22.7)	7 (26.9)	29 (49.2)
**Which of the following hand hygiene actions prevents transmission of germs** ***to the patient*** **?**						
**Α.** Before touching a patient *(YES)*	748 (91.8)	0.031	111 (95.7)	564 (93.2)	26 (96.3)	47 (83.9)
**Β.** Immediately after a risk of body fluid exposure *(NO)*	87 (10.7)	0.581	11 (9.4)	69 (11.5)	1 (3.7)	6 (10.5)
**C.** After exposure to the immediate surroundings of a patient *(NO)*	621 (76.2)	0.347	86 (74.1)	474 (79.5)	23 (85.2)	38 (73.1)
**D.** Immediately before a clean/aseptic procedure *(YES)*	710 (88.0)	0.000	100 (86.2)	548 (91.5)	26 (96.3)	36 (69.2)
**Which of the following hand hygiene actions prevents transmission of germs** ***to the health-care worker*** **?**						
**Α.** After touching a patient *(YES)*	722 (88.6)	0.034	106 (92.2)	549 (91.7)	23 (88.5)	44 (80.0)
**Β.** Immediately after a risk of body fluid exposure *(YES)*	729 (89.4)	0.058	113 (97.4)	544 (91.3)	25 (96.2)	47 (87.0)
**C.** Immediately before a clean/aseptic procedure *(NO)*	222 (27.2)	0.010	47 (41.2)	153 (25.7)	7 (26.9)	15 (28.8)
**D.** After exposure to the immediate surroundings of a patient *(YES)*	659 (80.9)	0.007	100 (87.0)	503 (84.5)	22 (84.6)	34 (66.7)
**Which of the following statements on alcohol-based handrub and handwashing with soap and water are true?**						
**Α**. Hand rubbing is more rapid for hand cleansing than handwashing *(True)*	582 (71.4)	0.589	90 (75.6)	434 (70.9)	19 (70.4)	39 (66.1)
**Β.** Hand rubbing causes skin dryness more than handwashing *(False)*	251 (30.8)	0.120	45 (37.8)	187 (30.6)	7 (25.9)	12 (20.7)
**C.** Hand rubbing is more effective against germs than handwashing *(True)*	315 (38.6)	0.004	59 (49.6)	214 (35.0)	13 (48.1)	29 (49.2)
**D.** Handwashing and hand rubbing are recommended to be performed in sequence *(False)*	279 (34.2)	0.782	37 (31.1)	215 (31.1)	8 (29.6)	19 (32.8)
**What is the minimal time needed for alcohol-based handrub to kill most germs on your hands?** *(20 s)*	352 (43.2)	0.002	54 (46.2)	267 (44.5)	17 (63)	14 (27.5)
**30. Which type of hand hygiene method is required in the following situations?**						
**Α.** Before palpation of the abdomen *(Rubbing)*	471 (57.8)	0.722	76 (63.9)	346 (57.1)	18 (69.2)	31 (55.4)
**Β.** Before giving an injection *(Rubbing)*	461 (56.6)	0.003	83 (70.3)	322 (53.1)	21 (80.8)	35 (62.5)
**C.** After emptying a bedpan *(Rubbing)*	211 (25.9)	0.027	40 (33.6)	149 (24.5)	5 (19.2)	17 (30.4)
**D.** After removing examination gloves *(Rubbing)*	768 (94.2)	0.159	108 (90.8)	581 (95.7)	25 (96.2)	54 (96.4)
**Ε.** After making a patient’s bed *(Rubbing)*	336 (41.2)	0.796	47 (39.5)	255 (42.0)	8 (30.8)	26 (46.4)
**F.** After visible exposure to blood *(Washing)*	605 (74.2)	0.007	80 (67.8)	465 (76.7)	23 (88.5)	37 (66.1)
**Which of the following should be avoided**,** as associated with increased likelihood of colonisation of hands with harmful germs?**						
**Α.** Wearing jewellery *(YES)*	774 (95.0)	0.654	113 (96.6)	583 (96.2)	26 (96.3)	52 (92.9)
**Β.** Damaged skin *(YES)*	600 (73.6)	0.032	98 (85.2)	440 (72.6)	22 (81.5)	40 (74.1)
**C.** Artificial fingernails *(YES)*	641 (78.6)	0.257	113 (96.6)	566 (93.2)	26 (96.3)	49 (89.1)
**D.** Regular use of a hand cream *(NO)*	558 (68.4)	0.502	82 (71.9)	417 (68.7)	22 (81.5)	37 (68.5)
**TOTAL KNOWLEDGE MEAN % (correct)**		(61.0)	(64.5)	(61.7)	(65.4)	(59.0)
**SD**		25.89	26.70	27.71	30.08	23.96

Statistically significant differences across occupational groups were found in the following statements: (a) *Hand rubbing causes skin dryness more than handwashing Χ*^*2*^ (1, *n* = 815) = 4.8, *p* = 0.028), (b) *The HH method required before palpation of the abdomen Χ*^*2*^ (1, *n* = 807) = 5.3, *p* = 0.021) and (c) *The HH method required after visible exposure to blood Χ*^*2*^ (1, *n* = 806) = 5.0, *p* = 0.024).

### Levels of perception

The following results refer to the levels of perception of HCWs in relation to HH (Table 3). Specifically, the question: *In your opinion*,* what is the average percentage of hospitalized patients who will develop a HCAI (between 0 and 100%)?* was answered as «*I don’t know» by* 35.9% (*n* = 294). Among those who responded, by giving a percentage, the frequencies were as follows: 0–20% (*n* = 114), 21–40% (*n* = 145), 41–60% (*n* = 123), 61–80% (*n* = 117), 81–100% (*n* = 27).

It was also found that the largest number of participants offered the response that hospital-acquired infection affects patient outcome from a *high degree* (48.4%) to a *very high degree* (48.6%). Furthermore, the largest number of participants answered that the effectiveness of HH in preventing hospital-acquired infections ranges from *high* (33.5%) to *very high* (62.8%). Additionally, the largest percentage of participants answered that HH is from a *high priority* (35.5%) to a *very high priority* (50.1%) for patient safety (Table 3).

The responses to the question: *“In your opinion*,* how effective would the following actions be to improve HH permanently in your institution?”* which consisted of eight items (sub questions), with the majority of the participants responded with *“Effective”* or *“Very effective”.*

The 7-point scale question: *“In your opinion*,* how effective would the following actions be to improve hand hygiene permanently in your institution?”* has been grouped to: Not effective (1, 2), Effective (3, 4, and 5) and very effective (6, 7). The item: “*Patients are invited to remind HCWs to perform HH”* got the highest percentage (20.3%) among all others as a non-effective action. Similarly, we grouped the following 7-point questions: (a) “What importance does the head of your department attach to the fact that you perform optimal hand hygiene?” (b) “What importance do your colleagues attach to the fact that you perform optimal hand hygiene?” and (c) “*What importance do patients attach to the fact that you perform optimal hand hygiene?”* to No importance (1, 2), Moderate importance (3, 4, 5), High - Very high importance (6, 7). The options “Moderate” and “High to Very high” importance had the highest rates (Table 3).

Finally, the question *“How do you measure the effort required by you to perform good HH when caring for patients?”* which was also grouped similarly, 62.4% responded with some effort to a big effort, and 37.6% responded with no effort (Table 3).

**Table 3:** Levels of perception in health-care workers

Question (*N* = 820)		Very Low%	Low %	High %	Very High %
In general, what is the impact of a health care-associated infection on a patient’s clinical outcome?		0.4	2.7	48.4	48.6
What is the effectiveness of hand hygiene in preventing health care-associated infection?		0.5	3.2	33.5	62.8
		**Low** **%**	**Moderate** **%**	**High** **%**	**Very High** **%**
Among all patient safety issues, how important is hand hygiene at your institution?		1.5	12.9	35.5	50.1
In your opinion, how effective would the following actions be to improve hand hygiene permanently in your institution?		**Not effective** **%**		**Effective** **%**	**Very effective** **%**
Leaders and senior managers at your institution support and openly promote hand hygiene		3.1		22.5	74.4
The health-care facility makes alcohol-based handrub always available at each point of care		1.5		11.9	86.6
Hand hygiene posters are displayed at point of care as reminders.		5.0		26.6	68.4
Each health-care worker receives education on hand hygiene.		2.3		14.6	83.1
Clear and simple instructions for hand hygiene are made visible for every health-care worker.		2.2		19.6	78.3
Health-care workers regularly receive feedback on their hand hygiene performance		2.8		20.0	77.2
You always perform hand hygiene as recommended (being a good example for your colleagues).		1.5		17.0	81.5
Patients are invited to remind health-care workers to perform hand hygiene.		20.3		26.7	53.0
		**No Importance** **%**		**Moderate Importance** **%**	***High –Very high Importance*** **%**
What importance does the head of your department attach to the fact that you perform optimal hand hygiene?		10.2		38.6	51.1
What importance do your colleagues attach to the fact that you perform optimal hand hygiene?		8.6		46.4	46.1
What importance do patients attach to the fact that you perform optimal hand hygiene?		29.8		42.3	28.1
How do you consider the effort required by you to perform good hand hygiene when caring for patients		**No Effort** **%**		**Some Effort** **%**	**A big Effort** **%**
		37.6		33.9	28.5

Regarding the differences between HCWs, 98% of nurses responded that *in general*,* the impact of a HAI on a patient’s clinical outcome is High – Very High* whereas the rest HCWs responded in lower percentages *Χ*^*2*^ (3, *n* = 817) = 14.1, *p* = 0.003. The vast majority of Ward Assistants (91.5%) responded that a*mong all patient safety issues*,* HH is considered high to very-high Χ*^*2*^ (3, *n* = 814) = 45.1, *p* = 0.000.

As regard to the effectiveness of certain actions to improve HH permanently, the majority of physiotherapists (92.6%) responded that *each health-care worker receiving education on HH is a very effective action Χ*^*2*^ (6, *n* = 814) = 14.5, *p* = 0.024.

The results of the study also revealed that the HCWs who attended a HH training program the three previous years presented with higher rates on the following questions:

- *Among all patient safety issues*,* how important is HH at your institution?* The majority *(*88.3%) responded as High – Very high priority, *Χ*^*2*^ (1, *n* = 814) = 3.9, *p* = 0.047.
- *In your opinion*,* how effective would be to improve HH permanently in your institution if each health-care worker received education on HH?* The majority (86.1%) responded as Very effective, *Χ*^*2*^ (3, *n* = 814) = 45.1, *p* = 0.000.
- *What importance does the head of your department attach to the fact that you perform optimal HH?* The majority (39.7%) responded as Very high Importance *Χ*^*2*^ (4, *n* = 816) = 17.8, *p* = 0.001.

It was also found, through an ANOVA test, that there were statistically significant differences in response rates regarding hand rubbing and handwashing between the various groups of HCWs F (3,521) 6.410, *p* < 0.001. Ward Assistants had higher response rates (M = 82.11, SD = 19.90).

### Perceived barriers to compliance to WHO hand hygiene guidelines

Several barriers to compliance were identified. Specifically, participants reported the highest percentages on the following barriers: (a) “The lack of necessary antiseptic preparations” as a “Very -“Extremely important” barrier (79.7%, M = 4.23, SD = 1.42), (b) “Workload” (72.5%, M = 3.92 SD = 1.15) as a “Very” -“Extremely important” barrier, c)“Indifference and/or negligence” as a “Very” -“Extremely important” barrier (72.2%, M = 4.01, SD = 1.23, d) “The illusion of glove protection” as a “Very - Extremely Important” barrier (71.6%, M = 3.97, SD = 0.98) and e) “Skin irritation from frequent hand washing without taking care with moisturizing lotion” as a “Very” -“Extremely important” barrier (60.6%, M = 3.73, SD = 1.07) and the HCWs’ lack of knowledge about the important contribution of HH on cross infection as a “Very” -“Extremely important” barrier (57.5%, M = 4.36, SD = 0.88). On the other hand, religious beliefs were reported as the highest percentage of a “Not - Somewhat important” barrier (53.6%, M = 2.53, SD = 1.76) (Table 4).

**Table 4:** Barriers to non-compliance to WHO hand hygiene guidelines

Barriers to non-compliance	Mean (SD)	Not -Somewhat important	Moderately Important	Very -Extremely Important
1. *Healthcare workers’ lack of knowledge about the important contribution of hand hygiene on cross infection*	4.36 (0.88)	92 (11.3%)	253 (31.2%)	488 (57.5%)
2. *The workload*	3.92 (1.15)	100 (12.3%)	122 (15.2%)	585 (72.5%)
3. *Shortage of staff*	3.73 (1.25)	224 (28.3%)	224 (28.4%)	342 (43.3%)
4. *Skin irritation from frequent hand washing without taking care with moisturizing lotion*	3.73 (1.07)	103 (12.8%)	215 (26.6%)	489 (60.6%)
5. *The lack of necessary antiseptic preparations*	4.23 (1.42)	50 (6.3%)	113 (14%)	641 (79.7%)
6. *The illusion of glove protection*	3.97 (0.98)	53 (6.6%)	175 (21.8%)	574 (71.6%)
7. *Patient’s needs come first*	3.45 (1.23)	174 (21.5%)	214 (26.6%)	418 (51.9%)
8. *The cultural background*	3.26 (1.33)	226 (28.1%)	208 (25.9%)	369 (46%)
9. *Religious beliefs*	2.53 (1.76)	431 (53.6%)	158 (19.7%)	214 (26.7%)
10. *Indifference and negligence*	4.01 (1.23)	115 (14.2%)	110 (13.6%)	583 (72.2%)

## Discussion

HCAI affect hundreds of millions of patients worldwide every year, leading to increased morbidity and mortality. HH is the most important, effective and simplest measure to prevent HCAI. Furthermore, good HH practices can prevent up to 15–30% of total HCAIs [5, 20].

Our study demonstrated that HCWs had a moderate mean percentage of knowledge on HH with an overall mean percentage score of 61%. In addition, significant and highly significant differences in knowledge were found among HCWs. Similar results had been obtained in previous studies which supported that knowledge of HH was necessary to improve HH practices among HCWs [21–25].

Inadequate knowledge about handwashing is a factor that can negatively affect handwashing behavior [26]. However, a study at Stanford University (USA) found that knowledge was not a significant predictor of HH behavior [27]. Attitudes toward HH are of utmost importance as they relate to knowledge about multidrug-resistant organisms [19], which is an emerging HCAI challenge.

The highest percentage of correct answers was among the physicians in our sample, followed by nurses and physiotherapists with regard to some questions, while the lowest percentage of correct answers was found in the group of ward assistants. These results may be due to the lack of ward assistant’s previous academic studies, but some other studies showed that perceived knowledge was higher among nurses than physicians [28, 29]. One may suggest that this discrepancy is due to prevention and control programs focusing mostly on nurses.

Regarding age groups, the results of our study showed that respondents over 41 years old gave correct answers at higher rates than the age groups below 40 years of age. This is consistent with similar, previous studies which reported that only the older participants had good attitudes and significantly better knowledge concerning HH [30–32]. However, Rajcevic et al. [33] found that knowledge and compliance rates were better in HCWs below the age of 40. This could be explained by regular training and practice sessions carried out by the institution as a part of their curriculum [29]. However, it was found that older participants (with more clinical experience) had better attitudes regarding HH. These contradictory results may be related to differences in the undergraduate curriculum which may have been modified to focus more on the prevention of HCAI and promotion of HH. It could be suggested that it would be better to examine the ancillary effect of older staff’s behavior concerning the views and knowledge level of other HCWs.

Regarding the effectiveness of formal HH training within the past three years, our results showed higher rates of correct answers from the HCWs who attended an infection control and prevention program that included HH when compared with those who did not, demonstrating statistically significant differences. These results contrast with the results of the study by Sopjani et al. [34], that showed that the level of knowledge of HCWs who had received formal training in HH was lower compared to those who had not. On the other hand, some studies [22, 24], presented similar results and they argued that there was no significant difference in the level of knowledge of participants who had received formal HH training and those who had not.

Regarding the effectiveness of HH in preventing HCAI and consequently improving patient safety, 33.5% of participants in our study answered that it is high and 62.8% very high. This is in agreement with the results of previous studies [35, 36], where the vast majority of participants considered HH as very effective in preventing HCAI and a powerful tool for patient safety.

Regarding the participants’ perception on the importance that their head of department gives to optimal HH, about half of the participants (51.1%) responded high to very high importance. These results are similar to those of Vikke et al. [37]. Some studies [26, 38] support that nurse leaders are role models for their subordinates and therefore by supporting optimal HH practices, increase HH compliance.

Regarding the differences between age groups, the study showed that participants over 41 years old had higher percentages for the effectiveness of HH in most questions. This result is also consistent with the study by Dreidi et al. [39] in which it was found that older participants (with more clinical experience) had better perceptions of HH. In contrast, Rajcevic et al. [33], found that HCWs under the age of 40 had higher levels of knowledge on HH and compliance compared to their older peers. These conflicting results may be related to differences either in their curriculum or the infection and control programs these individuals had attended.

More than 50% of respondents in our study identified the following six items as the most *“Extremely Important”* barriers: (i) the lack of necessary antiseptic preparations, (ii) workload, (iii) indifference and negligence, (iv) the illusion of protection from glove usage, (v) skin irritation from frequent hand washing without use of moisturising lotion and (vi) HCWs’ lack of knowledge about the important contribution of HH concerning cross infection. Religious beliefs were reported as the highest percentages of *“Not - Somewhat important”* barrier.

The two perceived barriers to compliance with the highest mean score were: HCWs’ lack of knowledge about the important contribution made by HH in preventing cross infection and the lack of necessary antiseptic preparations. In particular, 79.7% of participants reported the lack of necessary antiseptic preparations as a *“Very – extremely important”* barrier which was found in other studies as well [13–16, 25, 40, 41].

Furthermore, lack of knowledge about the important contribution made by HH in preventing cross infection, which got the second highest mean score among the perceived barriers to compliance, was defined as a “Very” to “Extremely important” barrier by 57.5% of participants. Several studies have shown that HCWs are unaware of the consequences of poor HH practices, including considerations like antibiotic resistance, length of hospital stay, hospital-acquired infections, and even fatality. In a study by Ahmadipur et al. [42], it is argued that the lack of awareness among HCWs’ has led to poor HH practices. Conversely, some studies have shown that HCWs have adequate knowledge of handwashing practices and awareness that unwashed hands are a major route of cross-contamination in hospitals [43–47]. Furthermore, other studies report that non-compliance with HH is not necessarily associated with HCW knowledge [48].

### Strengths and limitations

This study has both strengths and limitations. Its strengths include the fact that it was conducted in the largest hospital in Cyprus with a history of promoting good HH practices, and also it included the vast majority of HCWs in the hospital. As far as we are aware, this is the first study of its type in Cyprus, and so it is a baseline for future similar studies. Furthermore, our findings could help to shape future educational interventions based on identified needs.

However, this survey did not include other public or private hospitals, and the study’s response rate was only 60%. The inclusion of the HCWs of other hospitals either public or private would probably have improved our ability to make comparisons.

Potentially the inclusion of an intervention in the study could have facilitated exploring the measures needed to improve knowledge and perceptions of the WHO hand hygiene guidelines and perceived barriers to compliance. However, our aim was to capture the existing situation in order to describe a baseline for future studies.

## Conclusions

The study revealed that the level of perceived knowledge and perceptions on HH is moderate. Since HH in healthcare settings is a key component in reducing pathogen transmission and HCAIs, a strategy for multimodal HH should be adopted while taking into account local resources, administrative support, and training, focused on removing perceived barriers to compliance. Strategies aimed at changing the behavior of HCWs need to be part of the multi-stakeholder approach considering that behavior change is often complex. Finally, HH education not only increases knowledge, but also improves perceptions of the effectiveness of the actions to be implemented: but as literature revealed, HH training programs are not always successful and their impact is not always a long-term phenomenon.

## Data Availability

The data sets used and/or analyzed during the current study are available from the corresponding authors on reasonable request.

## Abbreviations

HH: Hand hygiene
HCWs: Health care workers
HCAI: Healthcare-associated infections
IPC: Infection Prevention and Control
AMR: Antimicrobial resistance
OECD: Organization for Economic Co-operation and Development
