# Correction: Health care workers’ knowledge and perceptions on WHO hand hygiene guidelines, and the perceived barriers to compliance with hand hygiene in Cyprus

**DOI:** 10.1186/s12912-024-02359-0

**Published:** 2024-09-27

**Authors:** Despo Constantinou, Ioannis Leontiou, Meropi Mpouzika, Koralia Michail, Nikos Middletton, Anastasios Merkouris

**Affiliations:** 1 Department of Nursing, Cyprus University of Technology Limassol Cyprus, Nicosia, Cyprus; 2 A&E Nicosia General Hospital, Nicosia, Cyprus

**Correction: BMC Nursing (2024) 23:644**

10.1186/s12912-024-02181-8

The original article erroneously displayed each author’s name in Family-Given Name order; this has since been amended.

